# Isolated Elastofibroma of the Thigh: A Case Report

**DOI:** 10.7759/cureus.42174

**Published:** 2023-07-20

**Authors:** Hüseyin Bilgehan Çevik, Ahmet Berkay Girgin, Aysun Gökçe, Burhan Kurtuluş

**Affiliations:** 1 Orthopedics and Traumatology, Ankara Etlik City Hospital, Ankara, TUR; 2 Pathology, Ankara Etlik City Hospital, Ankara, TUR; 3 Orthopedics and Traumatology, Diskapi Yildirim Beyazit Training and Research Hospital, Ankara, TUR

**Keywords:** pseudo tumor, soft tissue tumours, atypical location, thigh mass, elastofibroma

## Abstract

A 55-year-old female presented with elastofibroma of the thigh. On presentation, she complained of a palpable, painful mass on the anterolateral right thigh that had been present for one year. She had a history of surgery for a right femur fracture. On MRI, a soft-tissue mass was seen in the vastus intermedius muscle, as a heterogeneous lesion with streaky fatty and fibrous components. The fibrous component was isointense to the muscle, and the fatty component had a high signal on both T1- and T2-weighted images. Histopathological analysis after biopsy established the diagnosis of elastofibroma.

## Introduction

Elastofibroma is a benign soft tissue tumor, and it is histopathologically characterized by a mixture of collagen, elastic fibers, and adipose tissue. It is more commonly known as elastofibroma dorsi since it often occurs in the subscapular region. Although previously reported as a rare tumor, an autopsy study has shown a prevalence of one in four people in the elderly population [1]. There are several case reports of elastofibroma in locations other than the subscapular region [2-4]. We present a case of a female patient who had a history of femoral diaphyseal fracture 28 years prior and was diagnosed with unilateral thigh elastofibroma.

## Case presentation

A 55-year-old female patient presented with the complaint of a palpable, painful mass on the anterolateral right thigh for one year. The patient's history was remarkable for the open reduction and internal fixation with a plate and screws for a mid-1/3 diaphyseal fracture of the femur following a car accident in 1984. The implant had been removed one year after the surgery. Physical examination revealed scar tissue approximately 20 cm in length on the lateral thigh and a mass that was painful on palpation on the middle third right anterolateral thigh.

**Figure 1:**
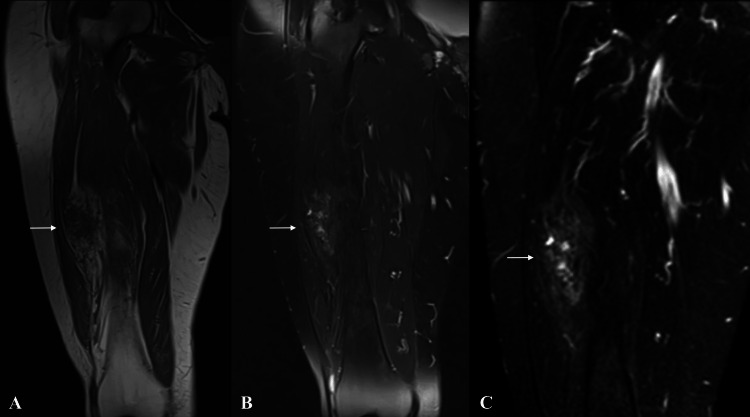
MRI of the patient (A) Coronal T1-, (B) T2-, and (C) gadolinium contrast T2-weighted images showing a relatively well-defined mass in the vastus intermedius (arrow). The lesion is streaky with lines of fatty and fibrous tissue MRI: magnetic resonance imaging

The radiographs showed cortical irregularities and thickening of the femur correlating with the history of fracture surgery. MRI showed an unencapsulated mass adjacent to the middle third of the femur with relatively well-defined margins. On T1 and T2 images, the tissue was seen to have fatty and fibrous components like tiger stripes. On gadolinium contrast T1 images, there was a heterogeneous low level of enhancement (Figure 1). The lesion was 75 x 32 x 29 mm in dimension.

**Figure 2:**
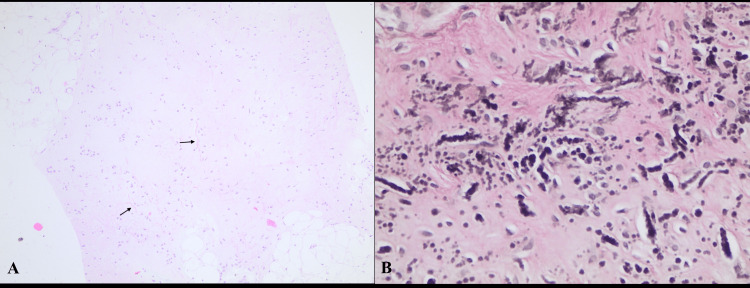
Histopathological analysis (A) Numerous eosinophilic altered elastic fibers in fibrous tissue (arrows, hematoxylin-eosin). (B) EVG stain highlighting the characteristic wavy, fragmented, coarse elastic fibers with serrated edges EVG: Verhoeff elastin stain

Tru-cut biopsy was performed for the histopathological diagnosis of the mass. The histopathological analysis revealed that the majority of the biopsy sample consisted of striated muscle tissue. There were also abundant abnormal eosinophilic elastin fibers admixed with collagen and mature adipose tissue. Wavy, fragmented, coarse, elastic fibers with serrated edges were observed with Verhoeff elastin stain (EVG) (Figure 2).

A diagnosis of elastofibroma was made based on the clinical, radiological, and histopathological findings. The diagnosis was discussed with the patient. Based on the literature search, an MRI of the contralateral thigh and subscapular regions was performed, and no lesions were observed; hence, it was decided to implement a conservative follow-up.

## Discussion

Elastofibroma is a benign soft-tissue tumor characterized by fibroblastic/myofibroblastic differentiation as per the World Health Organization soft-tissue tumor classification [5]. Although it has been occasionally associated with environmental and genetic causes, the exact etiology of elastofibroma is unknown [6]. Along with possible mechanical factors, the activation of CD-34 in mesenchymal cells has also been investigated [6]. However, it is thought that there may be repetitive trauma in the etiology, as it is often seen in the right subscapular region of women who are engaged in repetitive work [1]. In the present case, the patient's history of femoral fracture surgery suggests a possible traumatic origin of the elastofibroma.

Elastofibroma is frequently seen in the subscapular area and is uncommon in other regions [1-4]. According to the literature, elastofibroma has been reported as predominantly unilateral in some series and predominantly bilateral in others [6-9]. Elastofibroma is generally asymptomatic and detected incidentally on imaging performed for other purposes. When symptomatic, it may cause pain, and mechanical symptoms including snapping, clicking, or clunking, especially in the subscapular region. In the present case, the elastofibroma was localized to the thigh and was painful.

Radiological examination is an important step in the diagnostic process. On ultrasonography, elastofibroma appears as a lesion containing fatty tissue, and streaky collagen or elastic fibers with a relatively well-defined margin [7]. Radiographs may show a non-specific soft tissue mass or density. On MRI, although the borders of elastofibroma are relatively well-defined, no capsule can be identified. MRI can show a heterogeneous lesion with streaky fatty and fibrous components. The fibrous component is isointense to the muscle, and the fatty component has a high signal on T1- and T2-weighted images [10]. On gadolinium contrast T1 images, it shows a heterogeneous low level of enhancement [11]. Typical imaging features may eliminate the need for biopsy, especially for lesions in typical locations [12]. The MRI of the present case showed the typical streaky pattern of fatty and fibrous components. However, elastofibroma was not considered in the differential diagnosis of MRI because the localization was very atypical, and the MRI report indicated fatty infiltration and muscle atrophy.

For lesions that do not have typical imaging features, a definitive diagnosis can be reached with histopathological examination. The diagnosis of elastofibroma is significant because malignant tumors are included in the differential diagnosis [13,14]. Along with this, probably the most important differential is fibromatosis [6]. Moreover, the authors recommend a biopsy in order to make a definitive diagnosis and to avoid undertreatment or overtreatment [15]. Histopathologically, elastofibroma shows increased collagen fibers, spindle-shaped mesenchymal cells, and adipose cells following H&E (hematoxylin and eosin) staining. Multiple elastic fibers are also visible after EVG staining. Since most elastofibromas are diagnosed incidentally, and there are no known cases of malignant transformation, conservative treatment may be preferred, especially for asymptomatic patients [12]. In symptomatic cases, marginal excision is the choice of surgery since the risk of local recurrence is very low [8,16].

## Conclusions

We described a common case of elastofibroma with an atypical location. Despite the fact that imaging studies can establish the diagnosis of elastofibroma with high accuracy, a histopathological examination may still be required for a definitive diagnosis, especially for tumors with an atypical location.
